# Medical Opinion and Movement

**Published:** 1911-03-18

**Authors:** 


### Medical Opinion and Movement.

### Palpable Arteries in Children.

In the *Münchener Medizinsche Wochenschrift*, Dr. Hamburger draws attention to a curious phenomenon he has observed in certain children. Under normal conditions the walls of the superficial arteries, such as the radial or temporal, cannot be felt on palpation; one only feels the pulse, that is, the contraction of the artery, but not the arterial wall itself. In certain cases, however, the author states that he has been able definitely to feel the walls of the temporal and radial arteries and to convince himself of a certain degree of rigidity of the walls. He is of opinion that this rigidity is in no way of the nature of an arterio-sclerosis, but of a spasmodic kind, as it is very variable and may be accentuated at one moment and disappear entirely the next. He suggests that it arises from a certain tonicity of the vascular musculature and is a manifestation of a general neurotic or neurasthenic condition. Most of the cases were children of an irritable temperament, complaining frequently of headaches, palpitations, and the like. Other evidences of an exaggerated vaso-motor irritability were generally present; sudden flushing or pallor of the face, cold extremities, frequent sweatings, dermographism, and irregular respiratory pulse.

### Gastric Ulcer.

The modern medical treatment of gastric ulcer has resolved itself largely into a question of diet. Formerly milk was relied upon as the chief means of sustenance, being considered the most easily digested and therefore ensuring the greatest amount of rest to the stomach. More recently, however, diets consisting of eggs and fat in the form of cream, butter, or olive-oil have been advocated by preference, and definite lines have been laid down by different authors as to the manner and quantity in which these aliments should be given, and accordingly we have the diet of Lenhartz, the diet of Leube, etc. The diet of Yarotzky has now to be added to the list. Professor Yarotzky, of Youriev, feeds his patients suffering from gastric ulcer entirely on the whites of eggs and olive-oil. He considers milk or cream too heavy and gives the preference to white of egg, because it has been shown to pass rapidly from the stomach to the intestine. Fat inhibits the secretion of gastric juice and by opening the pylorus freely allows the bile and pancreatic juice to flow back into the stomach and neutralise the stomach contents. All this has been argued before, but the present author lays stress on the point of giving the eggs and oil at separate times, so as not to interfere with the digestion of the egg albumin by the oil. A further point in the treatment consists in the prohibition of water by the mouth as it excites gastric secretion. Fluid is supplied by means of rectal injections. For the same reason all forms of beef-tea, decoctions of gelatine, etc., are to be avoided. The details of treatment are as follows: In the case of a patient with gastric ulcer attended with hæmorrhage, on the first day the white of an egg is given in the morning and 20 grammes of olive-oil in the evening, and two or three rectal nutrient enemata during the day, or in severe cases nothing may be given by the mouth for the first two or thee days. Afterwards the number of eggs is increased daily up to eight, and the olive-oil by 20 grammes a day up to 120 or 140 grammes a day. As soon as the patient is taking several eggs a day the nutrient enemata are replaced by injections of sugar in water (10 grammes to 300 c.c. of water). The author admits that some patients are unable to take such large quantities of oil. In these cases he administers it by a tube.

### Anaphylaxis.

Drs. Minet and Leclercq, who have made a special study of the condition of anaphylaxis, find that the anaphylactic toxin is very fragile and can be destroyed, or at any rate disappears from a mixture of horse-serum and blood from a guinea-pig immunised against horse-serum by keeping it *in vitro* for six hours at the ordinary temperature of the laboratory. If the mixture is then injected into a fresh guinea-pig it is not sensitised passively, that is to say, the animal is not affected by an injection of horse-serum on the following day; but it is sensitised actively, that is, at the end of a fortnight an injection of horse-serum produces severe symptoms of anaphylaxis. The authors conclude from this that by keeping the mixture *in vitro* for six hours the toxogenin disappears, but the substance that excites active sensitisation remains. In the same way, by keeping a mixture of guinea-pig's blood immunised against diphtheria and anti-diphtheritic serum *in vitro* for six hours the anaphylactic poison is destroyed. The practical result of these findings is that if it is desired to re-inject an antigen or antitoxic serum into an animal already sensitised, as in the case of repeated injections of anti-diphtheritic serum in cases of diphtheria, in order to prevent the occurrence of anaphylactic symptoms, the animal or subject should be bled first to the amount of serum to be injected and the two mixed together in equal quantities and kept *in vitro* for six hours. If the mixture be then injected no anaphylactic symptoms should result.

### Adrenalin and Asthma.

The beneficial effects of the administration of adrenalin in attacks of asthma have been attested by several authors, and the treatment has already been dealt with in these columns. Hitherto the method advocated consists in the subcutaneous injection of a few minims of the adrenalin solution (1 in 1000) as soon as an attack of asthma develops. More recently, however, inhalations of adrenalin have been employed, apparently with equal success. Dr. J. Segel, of Vienna, combines the use of adrenalin with the administration of oxygen and places 1 c.c. of the 1 in 1000 adrenalin solution in the reservoir containing the oxygen. By experiments upon himself he first ascertained that inhalation of such a mixture of oxygen and adrenalin solution does not occasion any rise of blood-pressure. He publishes details of two cases, in which the attacks of asthma were extremely severe and persistent, and did not yield to other forms of treatment. The inhalations gave immediate relief and by repeating them at intervals for some time the attacks have ceased and the patients have completely recovered in general health. Dr. J. Pick, of Charlottenburg, has obtained similarly successful results by inhalation of powdered adrenalin through a specially constructed mask applied to the face. He also publishes an account of several severe cases of asthma which have rapidly yielded to this form of treatment. If the effect of the drug in these conditions is due to its local action on the respiratory mucous membrane, administration by inhalation would appear to be the more rational method, but this point has not as yet been clearly demonstrated. It would be interesting to compare the effects of the different methods, inhalation and injection, on the same patients, and in this way ascertain which is the more efficacious.

### Cocaine, Intravenously.

A contributor to the *Boston Medical and Surgical Journal*, Dr. Harrison, describes in detail some experiments which he made upon himself with the object of testing the practicability of using cocaine as a general anæsthetic by intravenous injection. He used a 2 per cent. solution, 4 drachms of which were slowly introduced into a peripheral vein; the time occupied over this gradual method was thirty minutes. The condition thus set up was then tested. A cut was made on the leg, right down into the fat, and was only very slightly felt. There was, in fact, analgesia everywhere; this had not entirely passed off in two hours, for a further cut was made to test the point. There was no loss of motor power at all; nor any inhibition of the cerebral centres, except that the author found it impossible to keep his mind on any one subject for more than a brief period. The experiment was carried out to determine whether the results indicated by animal experiment can be applied absolutely to man; it would appear that they cannot, for the anæsthesia produced was distinctly imperfect, while the very large dose employed is clearly one which is dangerous to human beings. The author is to be congratulated on his luck in escaping any serious consequences, and on his courage in so fearlessly risking them.

### Lupus of the Nose.

From the Finsen Institute at Copenhagen comes an account of a new method (invented by Dr. Pfannenstiel, one of the staff there) for treating lupus of the nose. The details are published in the *Berliner Klin. Wochenschr.*, and it seems that the theoretical basis of the treatment is as follows: Potassium iodide is given in liberal doses by the mouth, and it is believed that some of it is excreted by the nasal mucosa, with liberation of free iodine in the process. Whether this accounts for the symptoms of iodism or not, the author holds that the fact is established. This liberated nascent iodine is highly bactericidal, and its effect is aided by the presence of hydrogen peroxide, which is applied locally to the ulcers. Thus, also, more iodine is liberated than would otherwise be the case, and the potassium ion becomes transformed into caustic potash, which, again, has a deleterious effect upon micro-organisms. In the present report thirteen cases are tabulated, in all of which there was true lupus vulgaris of the nose (both internally and externally) and adjacent surfaces of the face. The diagnosis was established, in addition to the clinical evidence, by v. Pirquet's reaction; and syphilis was excluded by the Wassermann test—obviously an important precaution in view of the iodide treatment. The results as quoted are extremely good: in some cases improvement began at once, in others only after a time, and the permanence of the cure remains to be seen. Still it seems that this simple and effective method of treatment promises well and deserves attention.

### The Meiostagmin Reaction.

The present state of knowledge concerning the reaction which is known by this name is summarised in the *Interstate Medical Journal* by Dr. Juhnke. The principle of the reaction, it may be recalled, is that when a specific antigen is added to a specific serum, a lowering of surface tension results. This was first elaborated into a definite systematic reaction in Italy, by Ascoli and Izar. The reaction is positive in malignant disease in from 85 to 95 per cent. of all cases. Moreover, it is practically specific; for only one case has ever been reported in which a reaction has been found positive, after repeated trials, with a disease which, so far as can be determined, is not malignant. This patient is still living, so the point remains still unsettled. As much or even more can be said for the reaction in infectious diseases. No positive reaction is obtained with a specific antigen except in cases bacteriologically corresponding to the antigen. The time of appearance of a positive reaction remains to be further studied. It is relatively early in the acute infections, and in tuberculosis this is especially important: in experimentally infected animals it appears on the fifth day. In malignant disease it is still in doubt exactly at what stage the reaction may be expected to appear. Ascoli has found it in sixteen operable cases of carcinoma out of a hundred cases examined. Of these sixteen operable cases, four were quite early ones, in the third or fourth months of the disease.

### Blindness from Santonin.

That santonin is apt to produce untoward effects, including alterations in vision and especially xanthopsia is familiar enough; but the possibility of so small a dose as ½ grain being followed by blindness, apparently permanent, is probably not generally known, and although this untoward result is, it is to be hoped, very rare, it is one which should be borne in mind. A case of the kind has been recorded from East Africa by Dr. E. J. Baxter in a letter to the *Lancet*, in which he asks whether other practitioners have met with a similar case. Blindness from 5 grains has been recorded, but not, we think, blindness from $\frac{1}{2}$ grain. Dr. Baxter's patient was a girl about five years of age, who, suffering from both round worms and thread worms, was brought from the mountains for treatment, and a dose of santonin in castor oil was given, the whole being vomited almost immediately. No effect other than the vomiting having been produced by the dose, the child was brought for treatment again three days later, when $\frac{1}{2}$ grain of santonin was administered in 2 drachms of castor oil; no vomiting occurred this time, and eight hours after the administration of the remedy the bowels acted, and a large number of thread worms and a few round worms came away. The child then became very weak, and lay for two days as though she were dying, without moving her limbs or opening her eyes; on the third day she began to recover, and it was found she was quite blind. The parents, who lived twelve miles away up in the mountains, did not bring the patient to see Dr. Baxter again until three weeks later. She was quite blind, and the blindness was still complete six weeks afterwards.

### A Recovery from Sleeping-Sickness.

The *Bulletin of the Sleeping-Sickness Bureau* contains an account of a case of trypanosomiasis which ended in a cure. The patient, a Dr. Kérandel, was attacked by the disease in August 1907, a pseudo-furuncle appearing on the neck, along with continued fever lasting six days and nervous and cardiac excitement. For a month there were lighter attacks of fever separated by remissions of three to four days. At the end of this time characteristic symptoms of trypanosomiasis appeared. The organism was found in December and atoxyl treatment instituted, 1 gram being injected subcutaneously every ten days. Improvement followed for a time, but ten days after the third dose a relapse occurred. From December 1907, $\frac{1}{2}$ gram was given every five days, and improvement lasted for a month and a half; but as the result of a chill a relapse occurred and was followed by others at regular intervals. In the end atoxyl had no influence on the disappearance of the organisms. Intravenous injections of tartar emetic were started on September 14, 1908, and from that time parasites have never been found in the blood, and to-day the patient seems to have recovered completely. Tartar emetic by the mouth had no effect on the organisms, but he attributes his cure to the intravenous injections of the drug, of which he had four series made up respectively of seventeen, fifteen, fifteen, and eight doses daily of 10 c.g. tartar emetic. The first series caused him no trouble and he believes that sterilisation was effected after it. He bore the fourth series badly and it had to be discontinued after the eighth dose. He thinks anaphylaxis the cause of this intolerance.

### Serum Diagnosis of Whooping-Cough.

In 1906 Bordet and Gengou discovered the specific organism of whooping-cough, an ovoid body found in pure culture in enormous numbers in the treacheal and bronchial secretions at the onset of the disease. Owing to the appearance of other organisms it is extremely difficult to isolate the specific one at later stages, but information as to its presence can be obtained even then by the Bordet-Gengou reaction, known as the deviation or fixation of the complement. These authors have shown that the serum of children suffering from the disease contains antibodies to the microbe in question, or in other words that the reaction is positive when a culture of the specific germ is placed in contact with the serum of the patient. Quite recently Dr. A. Delcourt writes in the *Archives de Médicine des Enfants* that he has employed this reaction to discover cases of latent pertussis which occur at times during epidemics. His researches took place during an outbreak in a communal school at Brussels. He examined six children who coughed without exhibiting the characteristic crowing noise of the disease, and by means of the serum test found that they were suffering from pertussis. A similar result was noticed in the case of a female teacher who had been coughing for two months without showing the characteristic phenomena of whooping-cough. In another case, that of a pregnant woman, a nervous cough was found not to be pertussis, as the serum reaction was negative. It can easily be seen that the method will be of the greatest use in singling out those anomalous cases which by reason of their being unrecognised are such potent factors in the dissemination of the disease.

### Shock and Anæsthesia.

In the *Long Island Medical Journal* attention is drawn to two recent researches by American authors on the subject of surgical shock during administration of nitrous-oxide gas. Crile has made a number of experimental observations upon animals, from which he concludes that those anæsthetised with nitrous oxide resist traumatic shock much better than those anæsthetised with ether. In animals reduced by hæmorrhage, infections, hyperthyroidism, and the like, it was also found that "gas" has great advantages, and that the ganglion cells of the central nervous system show much less change subsequently. From clinical data he supposes that liability to infection is also lessened, and that post-operative neurasthenia is much less frequent. Henderson also holds that nitrous oxide anæsthesia is the best preventive of shock; this is the experimenter whose revolutionary theories as to what shock and its causation are have attracted so much attention. He favours gas and oxygen for major surgery, given by the method advocated by Gatch, who allows re-breathing for two or three minutes before a fresh supply of the gases is given. It is claimed that this causes increased depth of breathing, slowing of the pulse, and a rise of temperature and blood pressure.

